# CODA: an open-source platform for federated analysis and machine learning on distributed healthcare data

**DOI:** 10.1093/jamia/ocad235

**Published:** 2023-12-21

**Authors:** Louis Mullie, Jonathan Afilalo, Patrick Archambault, Rima Bouchakri, Kip Brown, David L Buckeridge, Yiorgos Alexandros Cavayas, Alexis F Turgeon, Denis Martineau, François Lamontagne, Martine Lebrasseur, Renald Lemieux, Jeffrey Li, Michaël Sauthier, Pascal St-Onge, An Tang, William Witteman, Michaël Chassé

**Affiliations:** Department of Medicine, Centre Hospitalier de l'Université de Montréal, Montréal, H2X 3E4, Canada; Faculty of Medicine, Université de Montréal, Montréal, H3C 3J7, Canada; Mila Quebec Artificial Intelligence Institute, Montréal, H2S 3H1, Canada; Department of Medicine, Jewish General Hospital, Montréal, H3T 1E4, Canada; Department of Emergency Medicine and Family Medicine, Université Laval, Québec, G1V 0A6, Canada; Department of Anesthesiology and Critical Care Medicine, Université Laval, Québec, G1V 0A6, Canada; Centre de Recherche Intégré pour un Système Apprenant en santé et Services Sociaux, Centre intégré de santé et de Services Sociaux de Chaudière-Appalaches, Lévis, G6V 3Z1, Canada; Centre de Recherche du Centre Hospitalier de l'Université de Montréal, Université de Montréal, Montréal, H2X 0A9, Canada; Centre de Recherche du Centre Hospitalier de l'Université de Montréal, Université de Montréal, Montréal, H2X 0A9, Canada; Mila Quebec Artificial Intelligence Institute, Montréal, H2S 3H1, Canada; Department of Epidemiology and Biostatistics, School of Population and Global Health, McGill University Health Centre, Montréal, H3A 1G1, Canada; Department of Medicine, Hôpital du Sacré-Coeur de Montréal, Montréal, H4J 1C5, Canada; Department of Anesthesiology and Critical Care Medicine, Université Laval, Québec, G1V 0A6, Canada; Centre de recherche du CHU de Québec-Université Laval, Université Laval, Québec, G1V 4G2, Canada; Centre de recherche du CHU de Québec-Université Laval, Université Laval, Québec, G1V 4G2, Canada; Centre de recherche du CHUS, Centre Hospitalier Universitaire de Sherbrooke, Sherbrooke, J1G 2E8, Canada; Centre de Recherche du Centre Hospitalier de l'Université de Montréal, Université de Montréal, Montréal, H2X 0A9, Canada; Centre de recherche du CHUS, Centre Hospitalier Universitaire de Sherbrooke, Sherbrooke, J1G 2E8, Canada; Centre de Recherche du Centre Hospitalier de l'Université de Montréal, Université de Montréal, Montréal, H2X 0A9, Canada; Faculty of Medicine, Université de Montréal, Montréal, H3C 3J7, Canada; Department of Pediatrics, Université de Montréal and CHU Sainte-Justine Research Centre, Montréal, H3C 3J7, Canada; Centre de Recherche du Centre Hospitalier de l'Université de Montréal, Université de Montréal, Montréal, H2X 0A9, Canada; Faculty of Medicine, Université de Montréal, Montréal, H3C 3J7, Canada; Department of Radiology, Centre Hospitalier de l’Université de Montréal, Montréal, H2X 3E4, Canada; Centre de Recherche Intégré pour un Système Apprenant en santé et Services Sociaux, Centre intégré de santé et de Services Sociaux de Chaudière-Appalaches, Lévis, G6V 3Z1, Canada; Department of Medicine, Centre Hospitalier de l'Université de Montréal, Montréal, H2X 3E4, Canada; Faculty of Medicine, Université de Montréal, Montréal, H3C 3J7, Canada

**Keywords:** machine learning, biomedical analytics, healthcare data management, distributed computing, federated learning, predictive models, resource usage analysis

## Abstract

**Objectives:**

Distributed computations facilitate multi-institutional data analysis while avoiding the costs and complexity of data pooling. Existing approaches lack crucial features, such as built-in medical standards and terminologies, no-code data visualizations, explicit disclosure control mechanisms, and support for basic statistical computations, in addition to gradient-based optimization capabilities.

**Materials and methods:**

We describe the development of the Collaborative Data Analysis (CODA) platform, and the design choices undertaken to address the key needs identified during our survey of stakeholders. We use a public dataset (MIMIC-IV) to demonstrate end-to-end multi-modal FL using CODA. We assessed the technical feasibility of deploying the CODA platform at 9 hospitals in Canada, describe implementation challenges, and evaluate its scalability on large patient populations.

**Results:**

The CODA platform was designed, developed, and deployed between January 2020 and January 2023. Software code, documentation, and technical documents were released under an open-source license. Multi-modal federated averaging is illustrated using the MIMIC-IV and MIMIC-CXR datasets. To date, 8 out of the 9 participating sites have successfully deployed the platform, with a total enrolment of >1M patients. Mapping data from legacy systems to FHIR was the biggest barrier to implementation.

**Discussion and conclusion:**

The CODA platform was developed and successfully deployed in a public healthcare setting in Canada, with heterogeneous information technology systems and capabilities. Ongoing efforts will use the platform to develop and prospectively validate models for risk assessment, proactive monitoring, and resource usage. Further work will also make tools available to facilitate migration from legacy formats to FHIR and DICOM.

## Objectives

Healthcare data are being produced at an ever-accelerating pace, yet most data remain in systems that are not interoperable.^1^ Centralized healthcare data repositories are cumbersome to develop and maintain and are usually created on an *ad hoc* basis to answer specific research questions. This approach requires meticulous prospective planning and complex legal agreements, which make it inflexible. This limits the quantity and quality of healthcare data available to researchers, clinicians, and decision-makers.

Distributed computation techniques have the potential to overcome some of the challenges associated with information technology (IT) resource centralization in healthcare.^2^ These methods involve dividing a computation problem into smaller parts and solving them concurrently in multiple nodes or systems. Federated analytics (FA) and federated learning (FL) are forms of distributed computation that allow researchers from different institutions to share non-disclosive insights about local data (eg, aggregate statistics, weights of predictive models) to partake in multi-site analyses (eg, meta-analysis, machine learning [ML] model training). These techniques have the potential to facilitate inter-institutional research collaborations by overcoming several of the administrative, ethical, and legal hurdles associated with data pooling, while providing good protection of patient privacy.^3^

The COVID-19 pandemic highlighted the inflexibility of the “pooled analysis” paradigm for knowledge exchange across healthcare institutions, which failed to provide the required flexibility to rapidly generate insights into an emerging public health threat.^4^ This spurred interest among several healthcare data stakeholders in Canada to evaluate existing libraries, frameworks, and initiatives aimed at facilitating FA/FL. A user-centered process was undertaken to understand the key constraints for the adoption of an FA/FL platform in our network of participating hospitals. Stakeholders included epidemiologists and clinical researchers from each institution, as well as machine learning researchers and practitioners, IT engineers, data security specialists, hospital administrators, legal specialists, and provincial government officials. Through this process, the following key design goals were identified:

- Support for both FL and analytical distributed computations.
- Interoperability with modern coding terminologies (eg, LOINC,^5^ SNOMED,^6^ ICD-10,^7^ CPT^8^) and healthcare data standards (eg, FHIR,^9^ DICOM^10^).
- Scalability to several million patient records while maintaining adequate performance.
- Ability to deploy and integrate new collaborating institutions within a short timeframe.
- Stringent disclosure control measures adapted for healthcare information
- Operability of the platform using exclusively non-commercial components.

Existing tools to facilitate FA/FL can be broadly divided into libraries (eg, TensorFlow Federated,^11^ Federated AI Ecosystem,^12^ Flower,^13^ PySyft^14^) frameworks (eg, SubstraFL,^15^ Fed-BioMed,^16^ OpenFL,^17^ and NvFlare^18^) and national or transnational initiatives (eg, German Cancer Consortium’s Joint Imaging Platform,^19^ Personal Health Train^20^) A summary of these tools, as well as their strengths and limitations, is provided in Table 1. Existing libraries and frameworks lack interoperability with modern healthcare data standards (FHIR, DICOM) or offer only partial support (eg, Reference 26) making multi-modal analyses difficult. Most use low-level orchestration protocols that are not amenable to auditing and make unrealistic assumptions about the flexibility of deployment environments in healthcare IT systems, increasing the time required to deploy at new institutions.^27^ One framework that is geared specifically toward the analysis of biomedical data lacks support for ML functionality, the addition of which would entail significant work, and is developed using an ecosystem of tools that is not widely used for performant web applications^28^^,^^29^ In most cases, the absence of integrated tools for no-code queries and data renders these tools inefficient for non-technical users; some libraries provide advanced security features at the expense of greater complexity and lower accessibility.^12^

**Table 1.** Related work: libraries, frameworks and platforms potentially applicable for distributed analysis of healthcare data.

Name	Description	Strengths	Open source	FHIR support	DICOM support	Explicit disclosure controls	Auditable communication channels and protocols	No-code data visualizations
Libraries								
Tensorflow Federated^11^	An open-source library developed by Google to facilitate machine learning in decentralized environments.	Facilitates decentralized machine learning, allowing for data privacy, and reduction of centralized server loads.	✓	✗	✗	✗	✗	✗
Federated AI Ecosystem (FATE)^12^	An open-source library designed to provide a secure computing framework for FL.	Emphasizes high performance and secure encryption methods like Homomorphic Encryption and Multi-Party Computation to ensure data privacy during the collaboration.	✓	✗	✗	✗	✗	✗
Flower^13^	An open-source library offering a flexible approach to FL compatible with various machine learning frameworks.	Supports multiple machine learning frameworks, and is scalable and adaptable for various FL setups.	✓	✗	✗	✗	✗	✗
PySyft^14^	An open-source library that extends PyTorch and TensorFlow to enable multi-party computations and FL.	Enables encrypted and privacy-preserving machine learning. Supports PyTorch and TensorFlow.	✓	✗	✗	✗	✗	✗
DataSHIELD^21^	A series of R libraries that enables the non-disclosive co-analysis of distributed sensitive research data.	Allows for the secure analysis of sensitive data without disclosure.	✓	✗	✗	✓	✗	✗
Frameworks								
Fed-BioMed^16^	A framework designed for FL in biomedical research, facilitating collaboration, and data sharing without compromising privacy.	Focuses on biomedical research, with features tailored to the specific needs of this field.	✓	✗	✗	✓	✓	**✗**
SubstraFL^15^	A distributed framework designed to facilitate collaborative machine learning projects.	Allows for multiple organizations to contribute to a shared model without directly exchanging their data.	✓	✗	✗	✗	✗	**✗**
OpenFL^22^	An open-source framework developed for FL.	Supports various deep learning frameworks, offering flexibility in model development and training across decentralized datasets.	✓	✗	✗	✗	✗	**✗**
NvFlare^23^	A framework from NVIDIA designed for FL, especially in the context of healthcare.	Performance of direct integration with NVIDIA ecosystem. Supports development in the healthcare domain.	✓	✗	✗	✗	✗	✓
Pathling^24^	A framework to derive simple analytics from FHIR data.	Focuses on biomedical research, with features tailored to the specific needs of this field.	✓	✓	✗	✗	✗	✓
National initiatives								
German Cancer Consortium Joint Imaging Platform^19^	A German initiative that establishes a distributed IT infrastructure for image analysis and machine learning across multiple hospital sites.	Facilitates collaboration and resource sharing among hospitals, enhancing research and treatment capabilities.	✓	✗	✓	✗	✗	**✗**
Personal Health Train^25^	An initiative aiming to provide a set of standards, guidelines, specifications, and reference implementations of the core components of the federated health data analysis.	Aims to standardize the approach to federated health data analysis, potentially simplifying collaboration and data sharing.	✓	✗	✓	✗	✗	**✗**

Considering the limitations identified in existing approaches, we developed the Collaborative Data Analysis (CODA) platform to satisfy the unmet need for a rapidly deployable, open-source package facilitating the ingestion, storage, analysis, and visualization of multi-modal EHR data, with the aim of achieving FA/FL. Our primary objective is to provide a descriptive analysis of the CODA platform and illustrate its FL capacities through an end-to-end simulation of multi-modal FL on FHIR and DICOM data. As a secondary objective, we aimed to assess the technological feasibility of deploying the CODA platform and scaling ingestion of data within real-world healthcare IT environments, across 8 hospital sites.

## Methods

### Platform architecture and implementation

**Figure 1.**
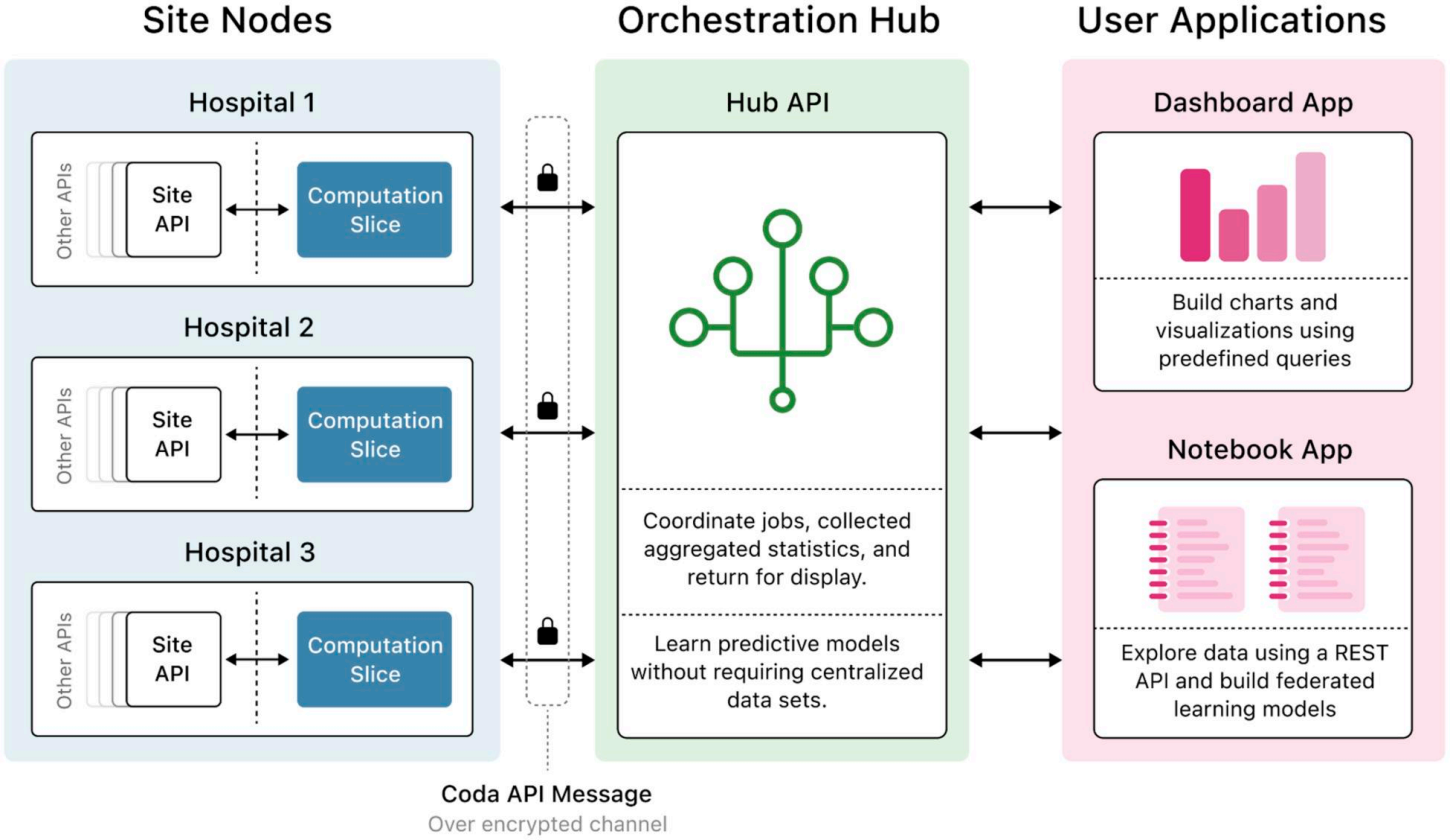
Schematic description of the microservice applications that communicate to enable distributed computation on the CODA platform.

CODA consists of several microservice applications that communicate to enable distributed computation on EHR data (Figure 1): a set of services that perform data ingestion and computation at each hospital site (site nodes); a system that coordinates local computations to complete distributed tasks (orchestration hub); and front-end components (dashboard and notebook applications) that allow users to launch custom analytical queries, generate data visualizations, and train machine learning models. A microservice architecture, as opposed to a monolithic structure, was selected to isolate heterogeneous, vendor-specific components behind standard interfaces and configuration parameters, enabling these components to be updated or changed with greater ease; to facilitate granular updates of different services on the site nodes; and to facilitate collaborative development of disjoint aspects of the platform by several participating teams.^30^

The site nodes (Figure 1, left panel) are located inside institutional firewalls and are deployed at each participating healthcare institution. They consist of storage and retrieval components for de-identified electronic health record (EHR) data, as well as imaging and waveform data. The site nodes additionally contain components that execute FA and FL tasks by obtaining limited subsets of data, as required for analysis. All communication channels between platform components communicate via fixed ports on TCP/IP and are protected using secure sockets layer/transport layers security. To enhance data privacy and observability of communications, no inter-site communication is permitted.

Data de-identification is performed before the ingestion of data into the site nodes. EHR data is stored in the Fast Healthcare Interoperability Resources (FHIR) format,^9^ while imaging and waveform data is stored in the digital imaging and communications in medicine (DICOM) format.^10^ The OMOP format was considered as an alternative to the FHIR format, yet we found that OMOP mappings did not exhibit the same level of granularity as the FHIR specification.^12^^,^^31^^,^^32^ For the same reason, we ruled against using the HDF5 and NIFTI formats to represent image data, noting that these formats can be trivially obtained from DICOMs, while the reverse is not true.

The orchestration hub (Figure 1, middle panel) is located outside institutional firewalls, does not have direct access to patient-level medical data, and can only obtain aggregate information (eg, mean, median, standard deviation, 95% confidence interval) or model weights (eg, ML model weights) by communicating with the site nodes. The hub coordinates distributed computations using a strict set of whitelisted operations, enforcing a standard message structure that is validated against an OpenAPI reference specification.^33^ Communication between the orchestration hub and site nodes is centralized through a single auditable WebSocket channel, minimizing the number of network port openings across the hospital firewall and providing a centralized auditable log to facilitate project oversight (Figure S3).

The 2 front-end applications (Figure 1, right panel) offer alternative methods to launch distributed computations and visualize results. The dashboard application allows non-technical users to perform FA queries by generating site-level and meta-analyzed statistical indicators in tabular and graphical formats. The notebook application comprises a Jupyterlab environment pre-configured with standard data analysis tools, allowing researchers and technical users to perform more advanced queries and train ML models using the Python programming language (version 3.8, Python Software Foundation, Python Software Foundation License).^34^^,^^35^

### Implementation details

Application containers were created using Docker (version 20.10, Mirantis, Apache License 2.0). For deployment in production environments, machines were configured and provisioned using Ansible (version 6.4.0, Red Hat, GPL License 3.0). For sandbox deployment and testing, containers were deployed and orchestrated using CapRover (version 1.10.1, Apache License 2.0). Access to these applications is restricted by a central identity management provider implemented using Keycloak (version 19.0.2, Apache License 2.0),^36^ which supports authentication with either OpenID Connect or SAML 2.0.^37^ Application components were developed in the TypeScript language (version 4.7, Microsoft, Apache License 2.0) running on Node (version 16.16, OpenJS Foundation, MIT License).^38^^,^^39^

### Data standardization and de-identification

Data standardization is critical to enable distributed computation across institutions with heterogeneous source systems. Within the CODA platform, tabular and numerical information is formatted according to the FHIR standard (version 4.0). FHIR data are stored in PostgreSQL (version 11.0, PostgreSQL Global Development Group, PostgreSQL License) via either DevBox or AidBox (version 3.7).^40^^,^^41^ Imaging and waveform data are stored in the DICOM format and served by Orthanc (version 1.10.1, GPL v3 License).^42^ Units of measure are expressed using Unified Code for Units of Measure (UCUM) standards, dates and times are stored in the ISO 8601 format, and country codes are represented in the ISO 3166 format. Table 2 shows a subset of common FHIR resources and highlights additional resource-specific coding standards that are preferred for integration with the CODA platform.

**Table 2.** FHIR resources and coding standards.

Type	Examples	FHIR resource	Coding standard(s)
Demographics	Age, gender, sex at birth, vital status, race, religion, marital status	Patient	HL7 CS
Past medical history	Past and new diagnoses	Condition	HL7 CS, ICD-10
Clinical encounters	Clinic or ED visit or hospital admission	Encounter	HL7 CS
Patient flows	Bed/unit arrival and departure time	Location	HL7 CS
Observations (clinical examination)	Weight, height, vital signs	Observation	LOINC
Observations (laboratory tests)	Biochemistry, hematology, serology, cultures, PCR tests	Observation	LOINC, SNOMED
Clinical interventions	Surgery, interventional radiology	Procedure	SNOMED, CPT
Medication history	Medications administered	Medication administration	AHFS
Laboratory tests	Biochemistry, hematology, serology, cultures, PCR tests	Observation	LOINC
Imaging tests	X-rays, CT scans	Imaging study	DICOM
Continuous signals	Electrocardiogram, arterial waveform	Observation	DICOM
All resource types	Date and time, country codes, units of measure		ISO 8601, ISO 3166, UCUM

Digital Imaging and Communications in Medicine (DICOM), International Classification of Diseases 10th Revision (ICD-10), American Hospital Formulary Service (AHFS), Current Procedural Terminology (CPT), Health Level 7 Coding Systems (HL7 CS), Logical Observation Identifiers Names and Codes (LOINC), and Unified Code for Units of Measure (UCUM).

Strict de-identification of all sensitive data is performed by each site before including patients in the CODA platform.^43^ Prior to de-identification, sites masked univariate patient characteristics that were shared by less than 5 individuals in the cohort or observation types (eg, a specific laboratory test) that were displayed by less than 5 individuals. Metadata were removed from DICOM files using a whitelist-based approach, where only fields confirmed as non-sensitive were included, and other fields were removed. Database keys are peppered and hashed using PBKDF2 with 100 000 iterations of SHA512 (1 pepper per row per site) and a secret salt (1 per site), each consisting of 128-character hexadecimal strings (512 bits). All secrets were generated using secure cryptographic random number generators.

### Distributed computations

The FA components of the CODA platform enable users to compute and compare aggregate statistical measures at the level of hospital sites, such as: “What is the mortality of ICU patients at hospital X?” CODA supports 2 high-level FA query types, within which resource selectors define the data scope of the query: (1) summarize query, which enables retrieval of record counts, as well as mean, standard deviation, and 95% confidence intervals (continuous data) or distribution mode (categorical data); (2) breakdown query, which enables retrieval of summary statistics on 1 variable partitioned according to a categorical variable (eg, creatinine according to sex) or a time interval (eg, patients alive over time). For breakdown queries, a minimum number of patients revealed in any individual data “bin” is enforced at the hospital site level. The FA dashboard additionally provides the ability to meta-analyze results. Meta-analyses are performed under a random effects model with inverse variance weighting.^44^ The CODA software development kit provides room for extensibility by incorporating additional FA procedures, subject to code review and approval by the project’s governance structure.

**Figure 2.**
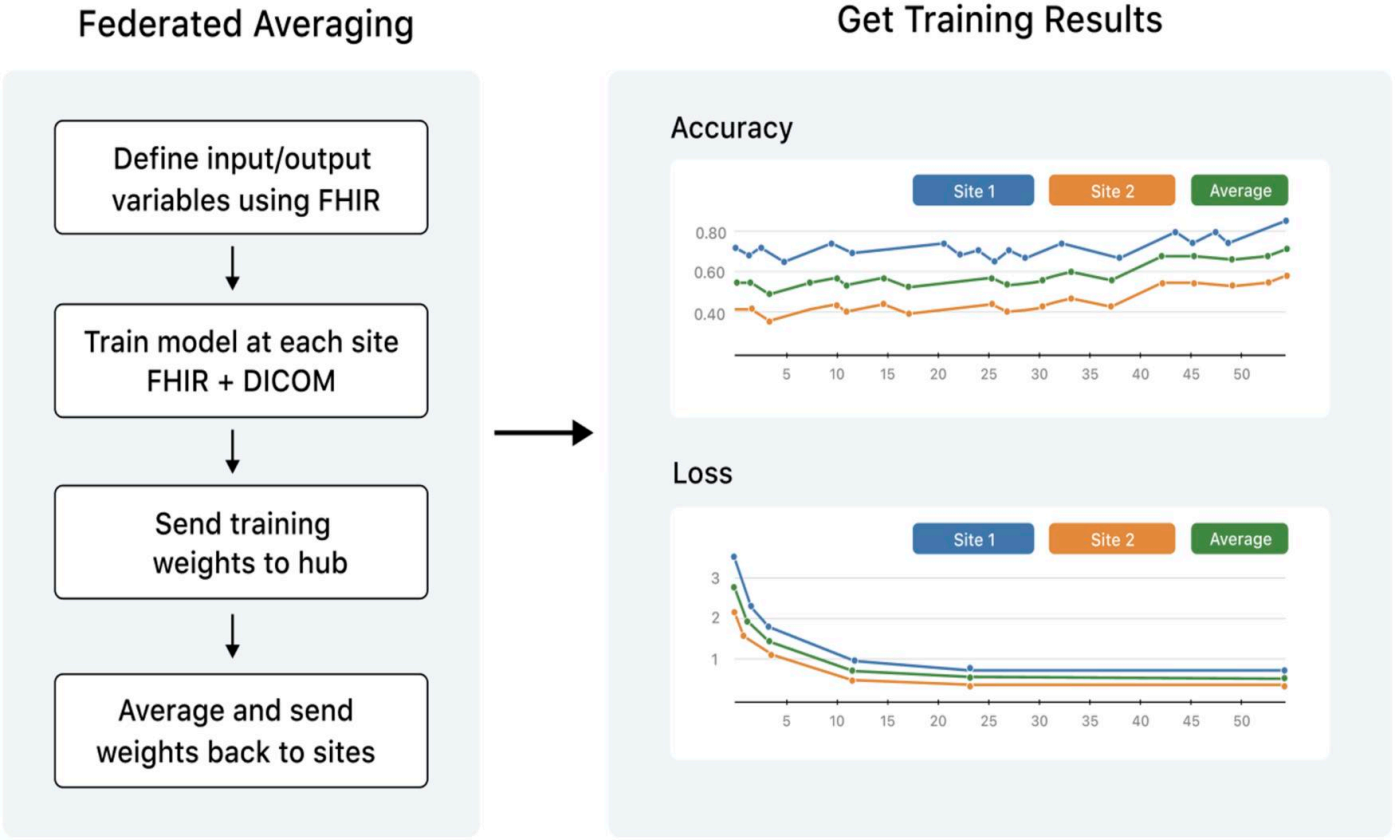
Illustration of federated training using the CODA platform. FL can be performed using FedSGD or FedAVG.

The FL components of the CODA platform enable users to train and evaluate ML models on multi-site data. ML functionality is supported by Tensorflow/Keras (version 2.0, Google, Apache License 2.0).^45^^,^^46^ Model architecture, training hyperparameters, and evaluation metrics can be specified using the Keras API. Distributed training can be performed using federated stochastic gradient descent (FedSGD) or federated averaging (FedAVG), as illustrated in Figure 2. Each site trains on its local data (FedSGD, *n *=* *1 epoch; FedAVG, *n *>* *1 epoch) and returns its weights to the hub.^47^ The hub then averages the model weights and passes this result back to the nodes as the input to the next training step. Pseudo-code for the FedAVG procedure is provided in Supplementary Material S1.

Given that the CODA platform is designed for exclusive use by authorized researchers operating under an REB-approved protocol, disclosure controls were aligned with a level of confidentiality that is appropriate for academic inter-institutional collaborations. As such, implementing algorithms with information-theoretical security guarantees (eg, differential privacy, secure multi-party computation) was not a design requirement. Ongoing work is exploring the applicability of differential privacy as a means to provide enhanced confidentiality protection in the event of accidental data disclosures.

### End-to-end testing of federated learning

To perform end-to-end testing of the CODA platform’s functionality, an online simulation environment was created with 4 servers representing hospital sites and 1 server for the orchestration hub (5 servers total). Each simulated site was assigned 1000 patients from MIMIC-IV,^48^ among the subset of patients with chest X-ray imaging data available from the MIMIC-CXR dataset^49^ (*n *=* *4000). The dataset was divided equally across simulated sites (*n *=* *1000 per site) and split as follows: 40% for training, 10% for validation, and 50% for testing. Inputs to the model were age, sex, a single laboratory parameter (pH), and baseline chest X-ray imaging. Output was in-hospital mortality as a binary variable. A simple multi-input deep neural network was created using convolutional layers for imaging data and fully connected layers for clinical data (Figure S2). The model was trained using stochastic gradient descent with the Adam optimizer, with a learning rate of 10^−5^, and binary cross-entropy loss.^50^ The performance of this model trained using FL (FedAvg with *n *=* *4 epochs per round) was assessed by examining loss, receiver-operating characteristic (ROC), and precision-recall curves.

### Technical feasibility study

A technical feasibility study was conducted by targeting deployment of the CODA platform across 9 public hospital sites in the province of Québec, Canada (Centre Hospitalier de l’Université de Montréal; McGill University Health Centre; Jewish General Hospital; Hôpital Sacré-Coeur de Montréal; CHU de Québec-Université Laval; Centre Intégré de Santé et Services Sociaux Chaudière-Appalaches; Centre hospitalier universitaire Sainte-Justine; Centre intégré universitaire de Santé et Services sociaux—Centre hospitalier universitaire de Sherbrooke; CIUSSS de l’Est-de-l'Île-de-Montréal). Participating sites aimed to deploy the CODA platform across all patients with potential or confirmed COVID-19 (defined as having undergone a PCR test for COVID-19).

Data-sharing agreements were established between participating sites to enable more flexible access as part of the platform’s initial development. A Governance Framework (Supplementary Material) was created to formalize the terms of collaboration between participating institutions. This framework sets forth guidelines regarding data ownership, safety controls, legal and technical responsibilities, and organizational structure when deploying a FL solution such as CODA. Projects utilizing data stemming from this feasibility study are overseen by a Governance Committee, which includes a senior representative from each partner institution. REB approval is required before platform access is granted for a specific project. Given that studies are performed via the secondary use of de-identified data, the CHUM REB granted a waiver for the need to obtain individual patient consent. Data collected in the CODA platform will be preserved for 10 years unless specified by an REB-approved study protocol.

### Role of the funding sources

The sources providing funding to the CODA project had no role in the design of this study, in the data collection, analysis, or interpretation processes, in the writing of the report, or in the decision to submit the paper for publication. The CODA project is a non-commercial endeavor, and all source code authored as part of the project is made available under the GNU General Public License, version 3.^51^

## Results

The CODA platform was developed between January 2020 and January 2023. Software code, documentation, and technical documents were released under the GPL v3 license (www.coda-platform.com). A set of standard FHIR templates were developed to assist users in migrating from legacy storage formats. An API Reference Specification was developed to guide the implementation of the various platform components. A Deployment Guide was created to facilitate the creation of sandbox/testing environments. A Data Security Framework was created to govern implementation practices relating to the authentication and authorization of users, as well as data protection at rest and in transit.

**Figure 3.**
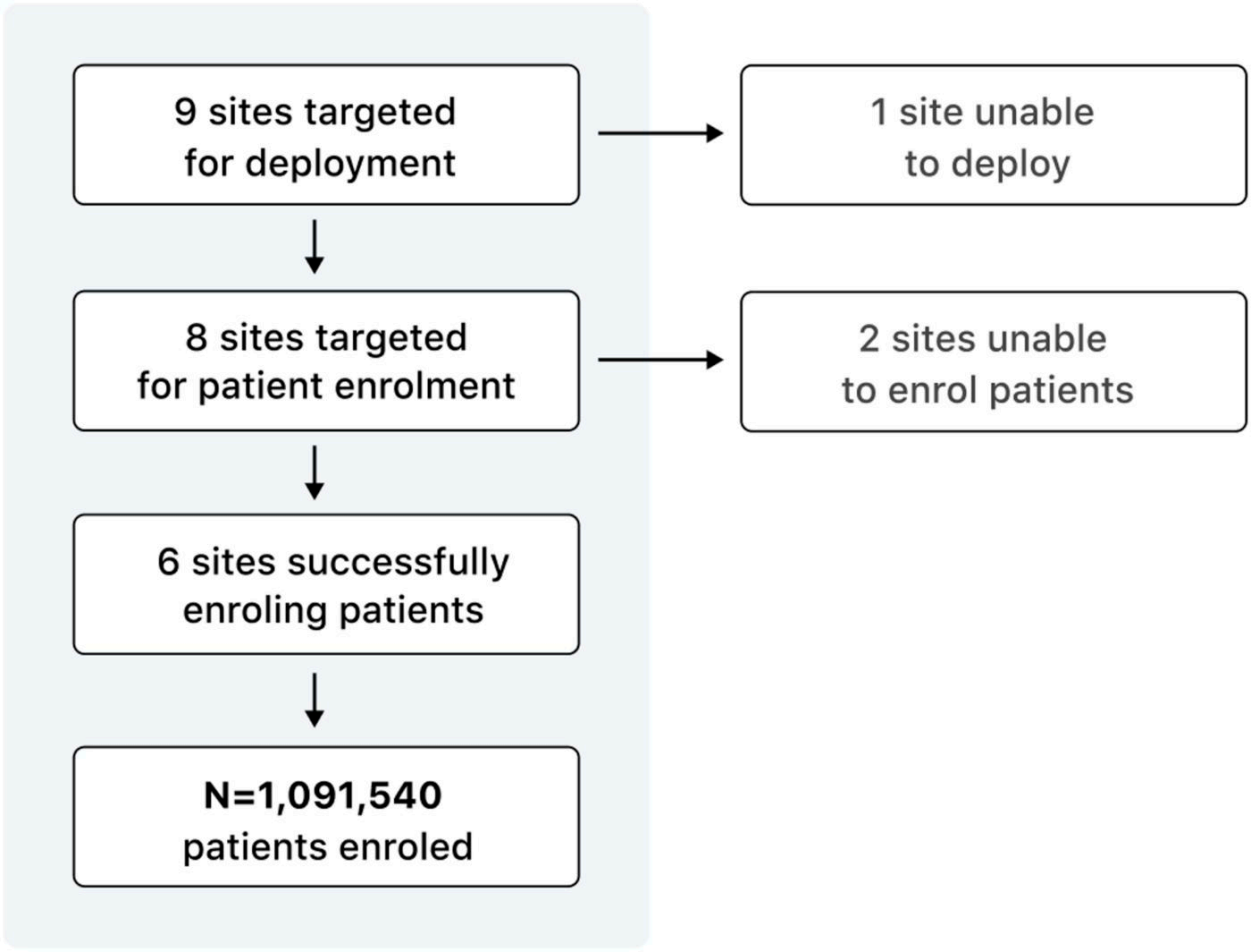
Key results from the CODA feasibility study, which aimed to deploy the platform across 9 public hospitals in the province of Québec, Canada.

Figure 3 illustrates key results from the CODA feasibility study, which aimed to deploy the platform across 9 public hospitals in Québec, Canada. A Governance Framework was created to formalize the legal and ethical terms of collaboration between participating institutions. Out of the 9 enrolled sites, 8 successfully deployed the platform locally and are connected to the CODA network. One site withdrew from the study before deployment started due to pandemic-related shortages in available IT personnel, and 2 sites have not yet provided patient data. As of publication, 1 091 540 patients have been enrolled in the CODA feasibility study cohort, totaling 46 181 904 FHIR objects and 3 777 716 imaging studies.

Table 3 presents a summary of the types and number of FHIR resources that have been mapped at each site at the time of publication. Sites were asked to provide FHIR objects for the “Patient,” “Encounter,” “Condition,” “Observation,” “MedicationAdministration,” and “ImagingStudy” resource types. Data mapping from older information systems to FHIR remains an ongoing process at most sites at the time of publication. A full descriptive analysis of the patient cohort is beyond the scope of this work and will be presented in a separate publication.

**Table 3.** Types and number of FHIR resources at each site enrolling patients, at time of publication.

Identifier	Patients enrolled (*N*)	FHIR types mapped (*N*)	Total FHIR objects (*N*)
Site 1	238 482	7/7	20 029 540
Site 2	92 852	5/7	991 671
Site 3	126 065	5/7	2 781 325
Site 4	557 082	5/7	5 701 307
Site 5	6985	1/7	6985
Site 6	7203	2/7	8890

**Figure 4.**
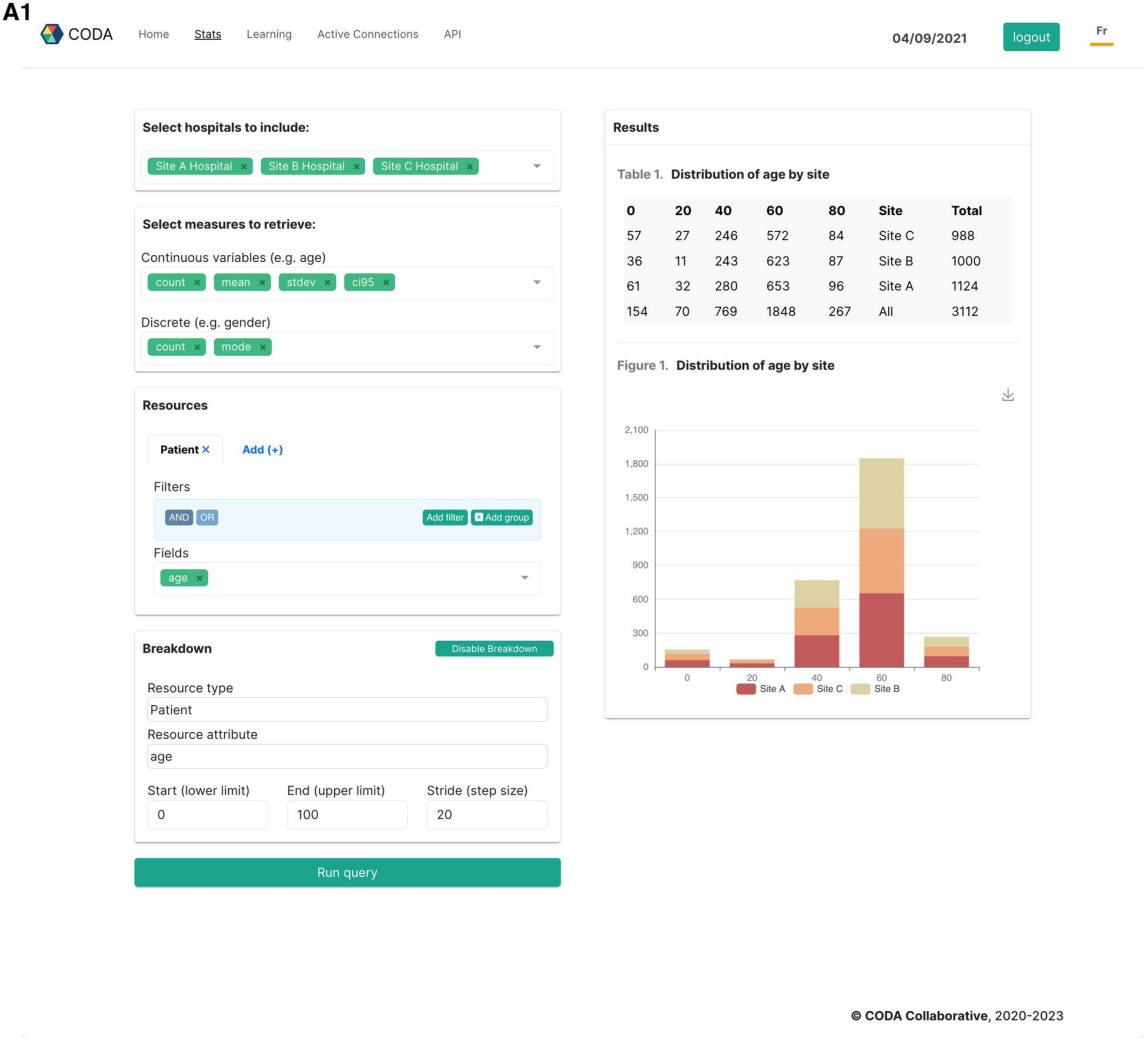

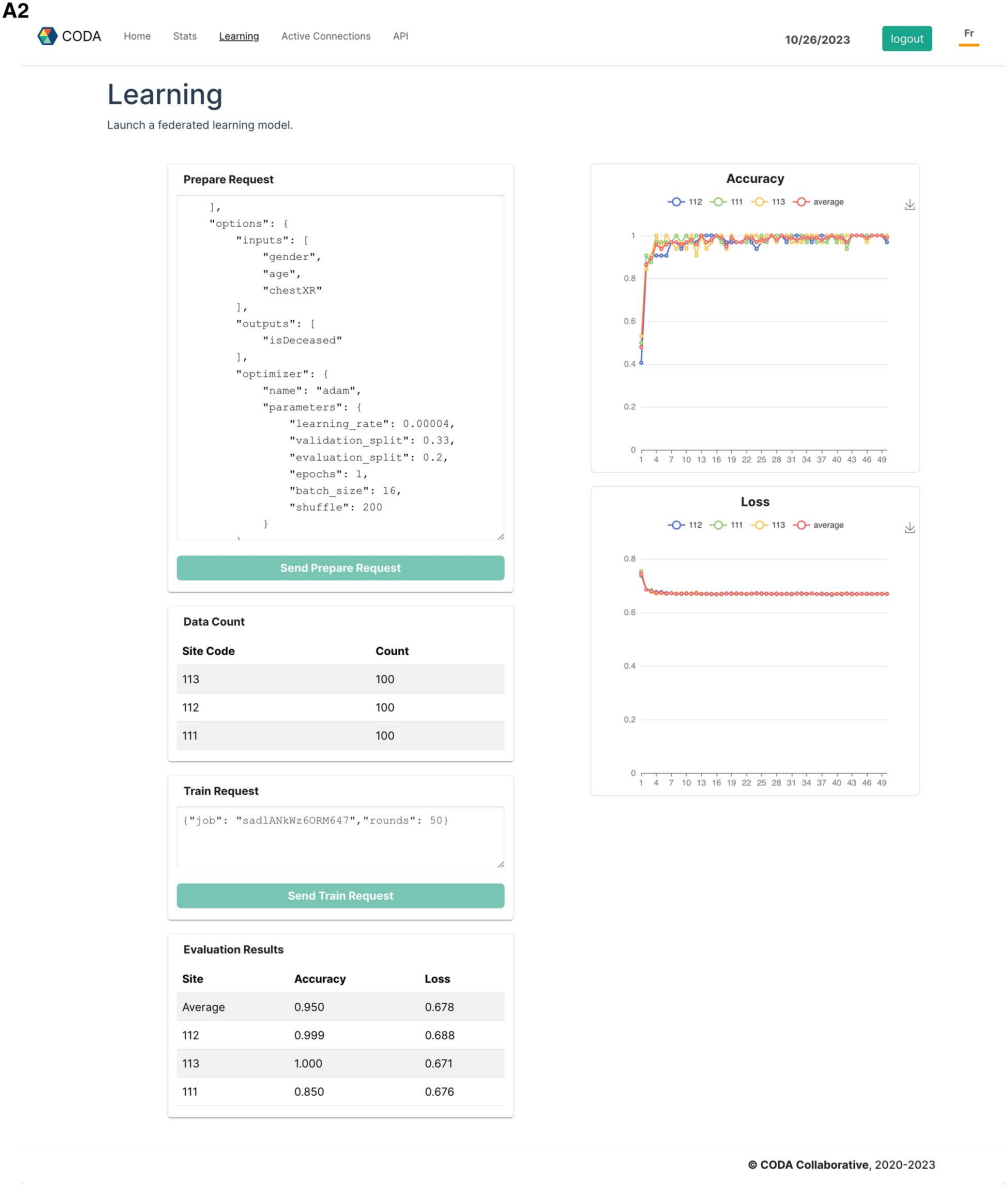

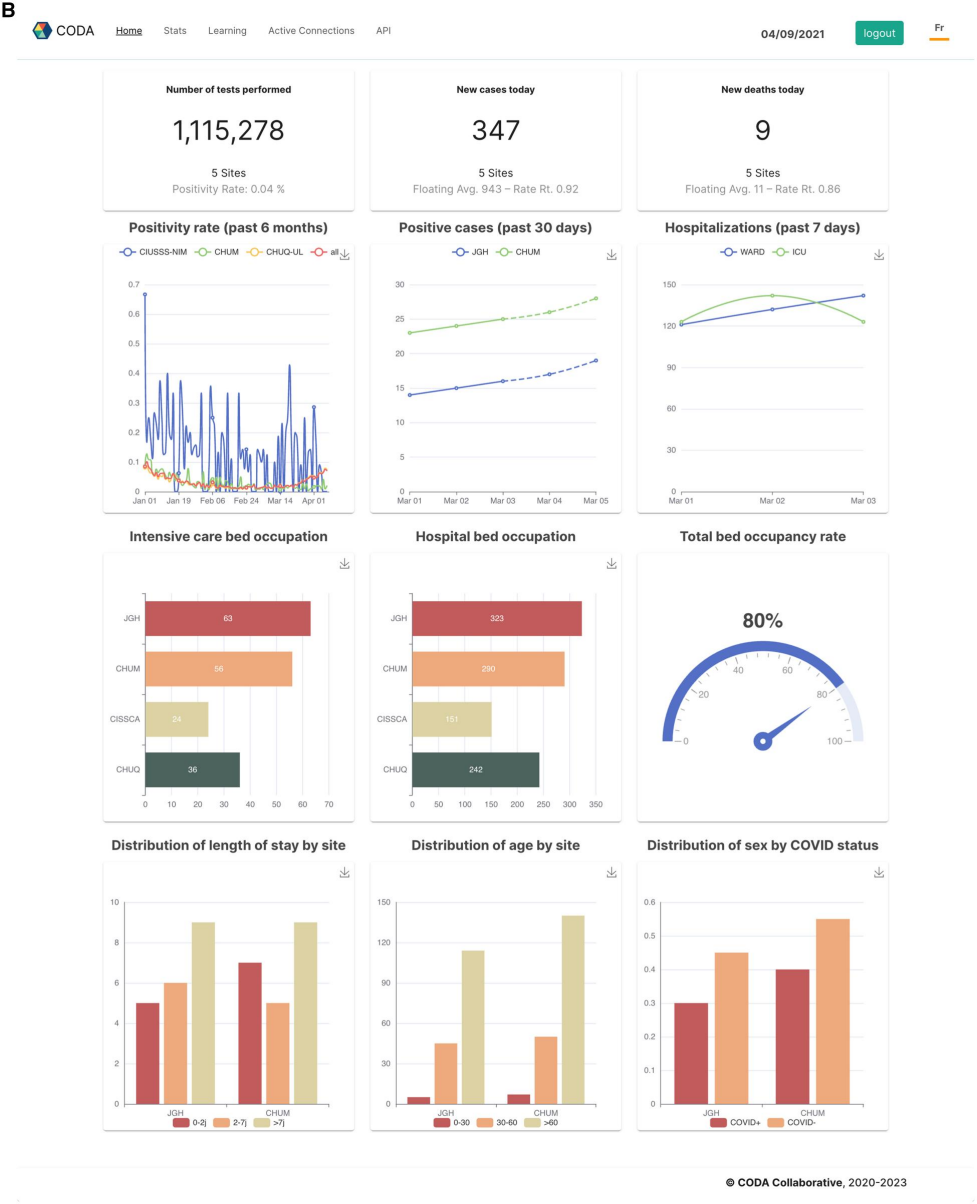
Screen captures illustrating practical applications of the CODA platform, such as creating custom multi-site data visualizations (panel A1), training FL models (panel A2), and building a real-time monitoring dashboard for COVID-19 (panel B).

A data visualization interface was deployed to demonstrate the translational potential of the platform by creating custom multi-site data visualizations (Figure 4A). Multi-site data visualizations were used to build a real-time COVID-19 monitoring dashboard, which was deployed across 3 hospital sites (Figure 4B).

Technical challenges relating to the deployment of the CODA platform’s software services and the ingestion of patient data are detailed in Table 4. The main challenge participating sites experienced was translating information from legacy database systems to the FHIR format and ensuring correct de-identification of EHR and imaging data. Several sites experienced difficulties allocating IT personnel to the project due to competing resource demands. The architectural decision to use a single channel for communication between the site nodes and hub was found to facilitate the acquisition of the required network permissions for deployment.

**Table 4.** Deployment steps and associated challenges experienced by sites participating in the technical feasibility study.

Deployment step	Associated challenges
Securing IT resources	Incentivizing local IT departments to allocate time dedicated to deploying and supporting the CODA platform. Identifying local mechanisms for acquiring, securing, and connecting hardware (GPUs) to enable FL. Identifying local processes for provisioning and accessing VMs.
Software installation	Obtaining required firewall permissions to access the source Docker images from the network environments of the allocated VM.
Software deployment	Obtaining required firewall permissions to connect to the CODA hub from the allocated VM.
Software maintenance	Obtaining the required accesses and approvals in order for technical staff to assist sites in fixing local bugs and issues. Fulfilling local institutional requirements for auditing of new software versions (eg, cybersecurity committees).
Data ingestion	Locating data in the relevant static source systems, and configuring a secure access point to read from the source systems. Identifying endpoints for streaming data sources (eg, HL7, FHIR), and configuring services to read from the data streams. Mapping data from legacy and/or proprietary formats to FHIR. Harmonizing terminologies between participating sites.
Data scalability	Obtaining local institutional permissions to reserve the disk space required in order to store a large number of patients. Avoiding strain on local hospital resources.

**Figure 5.**
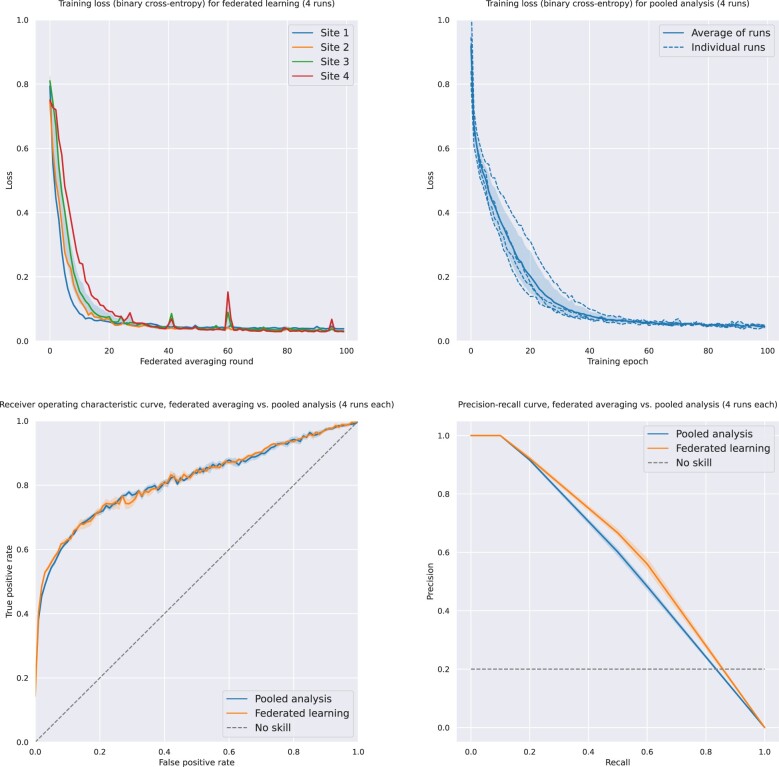
Results of training a multi-modality machine learning model via the CODA platform, performed on a sandboxed deployment with a subset of the MIMIC-4 and MIMIC-CXR databases.

Figure 5 illustrates the results of training a multi-modality machine learning model via the CODA platform, performed on a sandboxed deployment with a subset of the MIMIC-4 and MIMIC-CXR databases. The model consisted of a multi-input deep neural network, which takes in demographic variables (age, sex), laboratory values, and chest X-ray data and predicts in-hospital mortality as a binary variable. A schematic of the model’s structure is displayed in the Supplementary Material. Training curves, ROC, and precision-recall curves compare the model’s training behavior and prediction accuracy when trained using FL (FedAVG procedure) compared to offline learning performed on the entire pooled dataset.

The performance of the MIMIC mortality prediction model, as assessed by area under the curve (AUC) and *F*1 scores on a held-out test set (50%), is displayed in Table 5, according to the type of training procedure. As we aim to illustrate the FL functionalities of the platform rather than achieve state-of-the-art prediction accuracy, we used a simple model consisting of clinical, laboratory, and chest X-ray data. The model trained using FL had an AUC of 0.82 ± 0.01 as compared with 0.81 ± 0.01 for pooled analysis, while *F*1 scores were 0.58 ± 0.01 and 0.55 ± 0.01, respectively. Convergence was achieved after a similar number of epochs for both models (Figure 5).

**Table 5.** Comparative performance of mortality prediction models trained with FL versus pooled analysis on MIMIC-IV data.

Training method	AUC score (mean ± SD)	*F*1 score (mean ± SD)
Fed. Avg.	0.82 ± 0.01	0.58 ± 0.01
Pooled	0.81 ± 0.01	0.55 ± 0.01

## Discussion

This work has described the rationale, development, and deployment of CODA, an open-source platform for federated analysis and learning on distributed healthcare data. The applications of FA and FL in healthcare have been extensively reviewed elsewhere, and include resource management, risk stratification and prognostication, diagnostics and monitoring, disease understanding, clustering, and anomaly detection.^52^ Recognizing the potentially wide-reaching impact of facilitating these types of analyses, the CODA platform aims to simplify the multi-site analysis of healthcare data while protecting against unintended disclosure of identifying patient information. CODA is released under a fully open-source license (GNU General Public License, version 3) and made freely available to the research community.

The CODA platform was developed through a needs-based process and in consultation with relevant healthcare stakeholders, ensuring it addresses their unique needs and constraints. As such, it distinguishes itself from existing distributed computation frameworks in several ways. CODA is the first open-source FA/FL platform optimized for healthcare that features built-in combined support for the FHIR and DICOM formats and ontologies. This can facilitate research and extend the impact of FA/FL to non-technical users through no-code federated data visualizations. CODA provides fully auditable execution of distributed computations through a standardized API, enforcing explicit whitelisting of operations, which was a requirement for the healthcare use case. CODA was implemented using modern web standards and technologies to enhance maintainability and maximize project sustainability. It features a realistic deployment footprint (eg, 8 vCPUs, 16 GB RAM, and 512 GB disk) for institutions who can only dedicate a single VM to running the platform. Finally, CODA goes beyond providing a mere technological framework for distributed computation, putting forward a legal and ethical framework for multi-site collaborations involving FA/FL. We provide a standardized Governance Framework (Supplementary Material) that can help healthcare institutions start using these technologies more rapidly. This framework aligns with the principles of the Personal Health Train initiative, including responsible use of health data, ethics by design, and clear safeguards on data controls.^25^

Protection of patient confidentiality is of the utmost importance when performing distributed computations on healthcare data. The CODA platform is designed for end-users operating under authorization from an REB-approved research protocol. In this context, disclosing site-level aggregate statistics (eg, the mean age of patients admitted to the ICU at a given hospital) or disclosing ML model weights resulting from on-site training was considered ethically acceptable. There are also appropriate restrictions at the data retrieval layer. As such, initial work did not prioritize implementing algorithms with formal disclosure guarantees (eg, differential privacy, secure multi-party computation). Such algorithms can easily be implemented using the CODA platform and API, and reference versions are currently being developed.^53–55^

As demonstrated by the interim results of the feasibility study presented in this work, CODA was deployable across various technological environments in a public healthcare setting in the context of a worldwide pandemic. The scalability of the platform’s distributed data ingestion and statistical computation functionalities was demonstrated by creating a pandemic dashboard that showed real-time statistics for > 1M patients at 3 of the participating sites. The main technical challenge faced by participating hospitals related to the conversion of data stored in legacy formats to FHIR. In order to facilitate future deployments of the CODA platform, a tool is being developed to assist in aggregating disparate data sources and transforming them into FHIR format using Apache AirFlow (version 2.6.2, Apache Software Foundation, Apache License 2.0), while performing continuous quality controls on transformed data.

The multi-modality FL capacities of the CODA platform were demonstrated using clinical and imaging data from the MIMIC-IV/MIMIC-CXR dataset. A comparison of federated and pooled training approaches showed qualitatively comparable training dynamics and performance on a mortality prediction task, providing an end-to-end demonstration of CODA’s FL capabilities using a public dataset. It should be noted that the MIMIC-IV dataset was collected at a single hospital and lacks the heterogeneity required to perform a quantitative comparison of prediction performance between both approaches. As we made no hypotheses with regard to the comparative performance of both approaches on the MIMIC-IV dataset, a full hyperparameter search was not performed. The CODA platform is proposed as a tool designed to make research tackling these questions accessible to a wider range of practitioners. Ongoing work aims to further assess this using real-world multi-modal data from heterogeneous participating institutions.

The results of this work should be considered in light of the following limitations. First, this study described the core functionalities of the CODA platform using a single-center dataset. Assessment of training performance on larger and more heterogeneous datasets is forthcoming. Ongoing work continues to enrich the cohort with additional data sources and signals, implement more sophisticated FL algorithms, and further validate the platform on real-world and synthetic datasets. Second, a formal security audit of the initial public release of the platform has not yet been conducted, and the software is provided “as is.” Third, this study assumed that data would be de-identified and provided in FHIR/DICOM formats before inclusion in the platform. This assumes participating sites have the technical expertise to perform the required mappings and de-identification, which was not uniformly found to be the case in our feasibility study. Fourth, because of our mandate to protect privacy, this infrastructure does not solve the issue of linkage between site-level and out-of-site data sources. Finally, because only non-proprietary solutions were considered acceptable by our stakeholders, we did not formally assess or describe commercial solutions for FL on medical data.

## Conclusion

We demonstrated the functionality and deployability of a platform for federated analysis and learning on distributed healthcare data in a public healthcare system in Canada. The CODA platform facilitates CODA while maintaining strong disclosure controls and avoiding practical barriers to data sharing across sites. A Governance Framework was developed to help formalize the interinstitutional agreements required to participate in this type of distributed network. This platform will enable the development and study of new distributed computation techniques, as well as the prospective validation of epidemiological and ML models for individualized risk assessment, proactive monitoring, resource usage forecasting, optimization of healthcare delivery, and facilitation of multicenter large-scale clinical trials.

## Supplementary Material

ocad235_Supplementary_Data

## Notes

- Kevin Arsenault (Université Laval)
- Rima Bouchakri (Université de Montréal)
- Kip Brown (Université de Montréal)
- Rajeev Das (Université de Montréal)
- Adrien Dessemond (Université Laval)
- Alexandre Hamel (McGill University)
- Maxime Lavigne (McGill University)
- Bruno Lavoie (Université Laval)
- Tarek Lajnef (Université de Montréal)
- Julien Levesque (Université de Montréal)
- Louis Mullie (Université de Montréal)
- Jeffrey Li (Université de Montréal)
- Xuefei Shi (McGill University)
