# Supplementary material for: CODA: an open-source platform for federated analysis and machine learning on distributed healthcare data
Source: J Am Med Inform Assoc. 2023 Dec 21;31(3):651–65. doi: 10.1093/jamia/ocad235 (PMC10873779; doi:10.1093/jamia/ocad235)
Supplement: ocad235_Supplementary_Data [file ocad235_supplementary_data.zip › ocad235_Supplementary_Data/CODA_Manuscript-SupplementaryMaterial.pdf]

Supplementary Material

Figure S1. Network connectivity model for auditable communications.

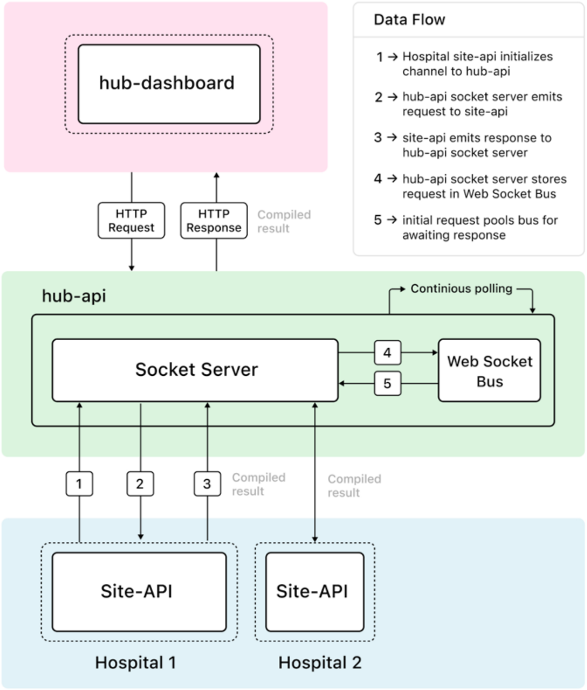

Figure S2. Pseudo-code for federated averaging procedure

Algorithm 1 Federated Averaging

Require: Number of clients K, number of local epochs E, batch size B, learning rate η, model w_0, and global rounds R.
Ensure: Trained global model w_R.

Initialize global model w_0.
for $r = 1, 2, ..., R$ **do**
  for each client k in C_k **do**
    Receive global model w_{r-1}.
    Initialize local model $w_{k,0} = w_{r-1}$.
    for $e = 1, 2, ..., E$ **do**
      Sample a mini-batch B of data from client k.
      Compute gradient $\nabla f_k(w_{k,e-1}; x_i, y_i)$ using mini-batch.
      Update local model $w_{k,e} = w_{k,e-1} - \eta \nabla f_k(w_{k,e-1}; x_i, y_i)$.
    end for
    Send updated local model $w_{k,E}$ to the server.
  end for
  Aggregate models from clients: $w_r = \frac{1}{|C_k|} \sum_{k \in C_k} w_{k,E}$.
end for
return w_R.

Figure S3. Multi-input neural network structure

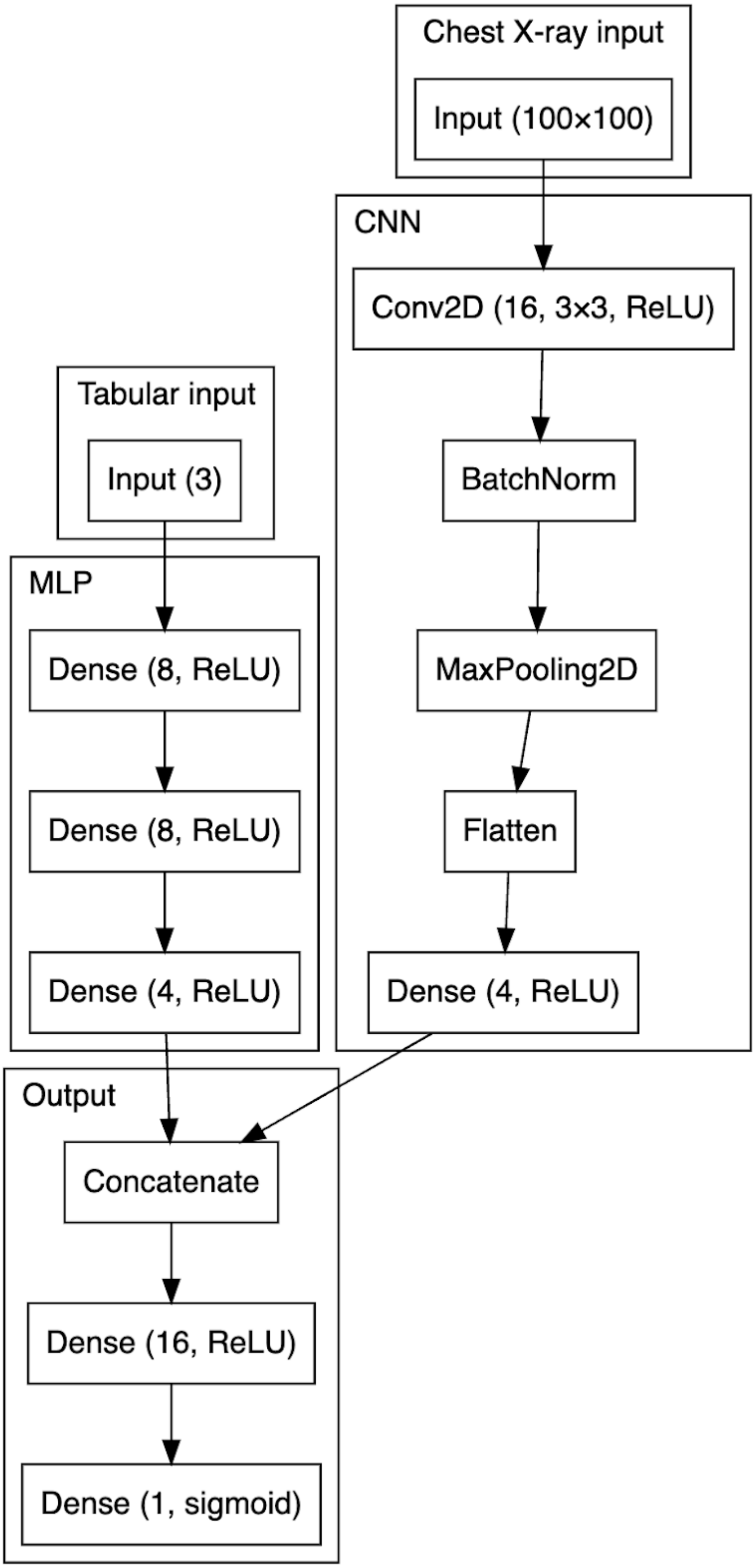

LaTeX code for Supplementary Figure S1

```
\documentclass{article}
\usepackage{algorithm}
\usepackage{algpseudocode}
\begin{document}
\begin{algorithm}[H]
\caption{Federated Averaging}
\label{algo:FedAvg}
\begin{algorithmic}[1]
\Require{Number of clients $K$, number of local epochs $E$, batch size $B$, learning rate
$\eta$, model $w_0$, and global rounds $R$.}
\Ensure{Trained global model $w_R$.}
\State Initialize global model $w_0$.
\For{$r = 1, 2, ..., R$}
\For{each client $k$ in $C_k$}
\State Receive global model $w_{r-1}$.
\State Initialize local model $w_{k,0} = w_{r-1}$.
\For{$e = 1, 2, ..., E$}
\State Sample a mini-batch $B$ of data from client $k$.
\State Compute gradient $\nabla f_{k}(w_{k, e-1};x_i, y_i)$ using mini-batch.
\State Update local model $w_{k, e} = w_{k, e-1} - \eta \nabla f_{k}(w_{k, e-1};x_i, y_i)$.
\EndFor
\State Send updated local model $w_{k, E}$ to the server.
\EndFor
\State Aggregate models from clients: $w_r = \frac{1}{|C_k|}\sum_{k \in C_k}w_{k, E}$.
\EndFor
\State \textbf{return} $w_R$.
\end{algorithmic}
\end{algorithm}
\end{document}
```

VIZ code for Supplementary Figure S2

```
digraph G {
    rankdir="TB";
    node [shape=rectangle];
    graph [fontname="Helvetica"];
    node [fontname="Helvetica", shape=rectangle];



    subgraph cluster_input1 {
        label="Tabular input";
        labeljust="l";
        myInput1 [label="Input (3)"];
    }

    subgraph cluster_mlp {
        label="MLP";
        labeljust="l";
        myInput1Dense1 [label="Dense (8, ReLU)"];
        myInput1Dense2 [label="Dense (8, ReLU)"];
        output1 [label="Dense (4, ReLU)"];
    }

    subgraph cluster_input2 {
        label="Chest X-ray input";
        myInput2 [label="Input (100×100)"];
    }

    subgraph cluster_cnn {
        label="CNN";
        labeljust="l";
        conv2d_1 [label="Conv2D (16, 3×3, ReLU)"];
        conv2d_1_bn [label="BatchNorm"];
        conv2d_1_bn_pooled [label="MaxPooling2D"];
        flatten [label="Flatten"];
        output2 [label="Dense (4, ReLU)"];
    }

    subgraph cluster_output {
```

```
        label="Output";
        labeljust="l";
        concat [label="Concatenate"];
        concat_dense [label="Dense (16, ReLU)"];
        output [label="Dense (1, sigmoid)"];
    }

    myInput1 -> myInput1Dense1;
    myInput1Dense1 -> myInput1Dense2;
    myInput1Dense2 -> output1;

    myInput2 -> conv2d_1;
    conv2d_1 -> conv2d_1_bn;
    conv2d_1_bn -> conv2d_1_bn_pooled;
    conv2d_1_bn_pooled -> flatten;
    flatten -> output2;

    output1 -> concat;
    output2 -> concat;
    concat -> concat_dense;
    concat_dense -> output;
}
```
