# Supplementary material for: CODA: an open-source platform for federated analysis and machine learning on distributed healthcare data
Source: J Am Med Inform Assoc. 2023 Dec 21;31(3):651–65. doi: 10.1093/jamia/ocad235 (PMC10873779; doi:10.1093/jamia/ocad235)
Supplement: ocad235_Supplementary_Data [file ocad235_supplementary_data.zip › ocad235_Supplementary_Data/CODA_Supplementary_GovernanceFramework.docx]

**CODA PLATFORM**

The CODA platform is distributed to each of the participating institutions. Each institution hosts a CODA server, which consists in several application components. Some components are used to load the institution’s data, others are used to store data following de-identification and standardization, and some are used analyze the de-identified and standardized data.

Each institution manages its CODA server and its content. It is responsible for the security surrounding access to the server, the security surrounding the loading and storage of its data in the local platform storage, and the terms of loading its data into the local bank of the platform.

**OWNERSHIP**

The implementation of the platform was made possible thanks to a close collaboration between the principal investigators (PIs):

- **[Name of site 1]: [Name of site PI]**
- **[Name of site 2]: …**

Since each institution is responsible for the extraction and collection of its data, each institution remains a trustee of its subset of data. The institution can conduct the desired and approved analyses as per local project approval mechanisms. The institutions to which the researchers are affiliated and the main researchers will remain trustees of all the material acquired as part of the collaboration. The obligations of each institution with respect to their data are defined by the law and remain unchanged by any activity performed in connection with the CODA platform.

The evaluating REB (Research Ethics Board) is the ethics committee of the research of the **[Name of central coordination center]** (Central Coordination Center).

**GOVERNANCE AND MANAGEMENT BODIES**

The entities involved in the governance and management of the platform are the PIs and the institutions to which they are affiliated.

**Participating Institutions**

The CODA project is developed in partnership with healthcare institutions. Once a member, these institutions have the following responsibilities:

- Outline a legal, ethical, and confidentiality plan for data management for their site;
- Establish a procedure for accessing the data of its institution;
- Identify a data trustee for its institution;
- Support the activities of data collection and extraction necessary to feed and maintain the local data bank;
- Guide the management of human resources required for data extraction and the maintenance of the data infrastructure;
- Disseminate the results of analyses performed using the CODA platform for the purpose of improving practices;
- Ensure the management of computer equipment hosting the components of the platform in accordance with internal rules and policies;
- Each institution, according to internal procedures and its local access committee, has the authorization to grant access to its institution's data.

**Executive Committee**

The researcher members of the CODA executive committee have the following responsibilities:

- Establish operating rules in compliance with the data management framework specific to each institution;
- Ensure that the rules and procedures stated in this framework and any policy or guideline developed by the participating sites are respected;
- Define the directions and development priorities of CODA;
- Establish the general rules for accessing the platform;
- Create a platform access committee and establish an independent, impartial, and conflict-free access process.

**Central Coordination of the Platform**

The coordination of CODA's activities as a whole is ensured at the Central Coordination Center by individuals who will ensure the coordination of infrastructure and partnerships, as well as ethical compliance and the ethical suitability of participating institutions. Their main functions are:

Common Functions

- Receive requests for access to the platform, present them to the central access committee of the CODA platform, and inform the applicant of the final decision as explained in annexes 4 and 5;
- Carry out occasional audits for each of the institutions in a process of support and improvement;
- Keep essential documentation up to date.

Coordinator of Infrastructure and Partnerships:

- Monitor the progress of the platform at the institutional level;
- Ensure links between institutions and the commercial and academic partners of the platform;
- Monitor the use of large-scale data;
- Monitor technology to identify improvements to be made;
- Keep the platform's technical documentation up to date (Deployment Guide, Security Guide);
- Establish contact with potential collaborators and formulate an expansion plan, if necessary.

Coordinator of Ethics and Suitability:

- Ensure liaisons with the research ethics committees (CÉR) of the institutions;
- Monitor the institutions' suitability evaluations;
- Write ethical documents and their modifications as required, according to the models and templates required by the Central Coordination Center;
- Submit and monitor all ethics submissions;
- Carry out the annual ethics renewal;
- Monitor contracts with the Central Coordination Center;
- Monitor the platform's finances.

Local Coordination of the Platform by the Participating Center:

The coordination of CODA's activities is ensured by a person in charge of local coordination for each institution, who has the main functions of:

- Collaborate, if necessary, with the central access committee in evaluating access requests for their institution;
- Guide users on access procedures;
- Keep a register of current accesses;
- Ensure the confidentiality of information, data, and the security of the bank, working hand in hand with their local access committee;
- Ensure that bank users comply with the rules set out in this management framework;
- Monitor the use of data;
- Act as the main point of contact for exchanges between the central coordination center and their institution.

**Platform Steering Committee**

The platform steering committee is composed of members of the executive committee as well as key individuals in the fields of machine learning, statistics, and applied human health data science. The functions of the steering committee are:

- Validate the strategic decisions made by the executive committee;
- Act as a facilitator for the platform's directions by sharing its vision;
- Suggest improvement paths, especially in terms of risk.

**Central Access Committee**

The central access committee is responsible for evaluating any requests for use of the CODA platform’s functionalities or data. The committee consists of:

- One representative from each participating institution;
- One member representing the fields of medical imaging, data science, machine learning;
- One member capable of conducting risk analysis for patient re-identification;
- Two platform coordinators who act as non-voting access officers.

The evaluation of requests is carried out in light of the internal policies of each participating institution, the risk of patient re-identification, and the evaluation of the algorithm or method proposed for analysis. The assessment may require exchanges, consultations, and verifications between the central access committee and the local coordination center of the institution for which access is requested.

**Patient Partner Strategic Committee**

The patient partner strategic committee is composed of patient partners and experts in partnerships with patients and the public. Its mission is to promote information, engagement, and effective participation of patients and the public in the decision-making processes of the CODA platform and in approved research projects to access institutional data. The strategic committee's main functions are to:

- Contribute to the establishment of evaluation criteria and guides to promote patient and public partnerships in research projects;
- Support the platform's decision-making bodies in evaluating research projects submitted for data access requests;
- Continuously accompany researchers in adopting best practices in terms of partnerships with patients and the public in research;
- Inform and engage the public in discussions and the valorization of research results stemming from data use.

**Internal Committee of the Participating Institution**

The internal committee is defined according to the need and internal policies of the institutions.

**DATA ACCESS AND REQUEST PROCESSING**

Participating institutions retain their prerogatives regarding access to data under their responsibility.

The initiator of the data access request to the platform must be a researcher with a "regular researcher" status (i.e., an individual with a valid researcher status). For projects from external academic environments, government agencies, or projects from the industry, collaboration with a local researcher prior to the access request is required. However, it is the responsibility of the institutions to establish their own local frameworks, rules, and data access procedures.

In the interest of transparency, any collaboration with the private sector will be disclosed on the platform's website. Only aggregated results will be shared with the private sector, and collaboration with an internal researcher or a department will be required.

**Access Authorization Process**

The process to allow access to the platform and controlled access data is initiated by an access request via a form that will be sent to the access officers of the central coordination center.

The researcher must submit an access request that includes a summary of the research project, a justification of the requested data, and proof of grant or availability of the funds necessary to carry out the project.

For each use request, access fees to the platform will be required and reflected in the access agreement.

The Central Coordination Center will undertake an initial review of the submission. Following this, the project will be submitted and analyzed by the Central Access Committee, which will relay its decision to the central coordination center.

Access requests will only be considered for research projects that meet the following conditions:

- The research project must have scientific value and be scientifically grounded;
- The research project must be presented and conducted by qualified researchers;
- The research project must be approved by a duly constituted REB for the expedited process, or for the regular process, no later than following the approval of the central access committee.

Once ethical approval has been obtained and access approved, an access agreement will be signed with the researcher using the data and their institution before final access authorization. This agreement includes:

- The fees for accessing the platform, as applicable.
- Measures in place to ensure confidentiality and security.
- The fact that models resulting from the use of the CODA platform must not be shared with unauthorized third parties.
- The fact that publications resulting from projects will be shared with the CODA platform team so that they can be incorporated into the platform's database.

Once the agreement is signed, the applicant must submit their code for a prototyping phase on simulated data.

The analysis phase on real data will follow the prototyping phase. Analyses will be carried out in accordance with the access agreement and the internal procedures of the institutions that approved the access request.

A follow-up of publications with the user-researcher must be ensured by the local coordination center.

**PRIVACY AND CONFIDENTIALITY PROTECTION**

The fundamental principle of the CODA platform is based on the absence of patient data exchange (individual data). In addition to control at the access management level, privacy and confidentiality protection is structured around the de-identification of extracted data and compartmentalized infrastructure.

Data De-identification:

All data stored in the CODA platform’s secure components is de-identified. They are linked via a unique random non-sequential identifier created and generated by the responsible administrator. This identifier is only accessible to members of the immediate local team regardless of research team permissions.

In addition to data de-identification, the bank establishes restrictive data sharing standards. On the one hand, all sensitive patient information leading to direct identification is removed, such as individual names or numbers (e.g., textual data contained in medical records, images, etc.). Moreover, masking of analysis results using the k-anonymization principle (k=5) is implemented, and free-text format data are prohibited. Finally, restrictions are placed on imaging data (DICOM) by whitelisting non-sensitive fields.

Compartmentalized Infrastructure:

The infrastructure in place allows for increased security through the separation of platform components that require access to different scopes of data. Access is restricted and is the responsibility of the staff of the participating institution identified by the lead researcher. Thus, de-identified and standardized data in FHIR format is stored in one platform component. Another platform component serves as a a computational unit and allows only temporary viewing of aggregated data or parameters resulting from the training of a model (weights). This infrastructure promotes the confidentiality of sensitive data through compartmentalization.

The federated model allows interoperability between the different participating institutions, and the independence of local data banks hosted in the different institutions promotes the principle of confidentiality. Moreover, only exchanges of weights or aggregated data will transit between the participating institutions. Thus, the infrastructure deployed within the participating institutions promotes the confidentiality of individual data, and the federated model allows only the sharing of aggregated data between the different institutions.

**DATA MANAGEMENT AND ACCESS SECURITY**

The servers dedicated to the CODA bank consist of a series of hardware servers on which a network of virtual machines is installed, used to extract, process, redistribute, store, and analyze data; all on storage spaces contained within the infrastructures of the participating institutions and connected via a central server. Only targeted users from each participating institution with the necessary privilege have access to the re-identification keys that allow matching between the platform's data and the data from local source systems.

Data and subsets thereof are stored on servers dedicated to the CODA platform and protected within local infrastructures. They are subject to the same security standards as patient data from hospitals. Only responsible persons (IT, security officer, etc.) at the infrastructure level of the institutions and server maintenance, through badge authentication, can physically access the secure space of their institution's servers.

Only the institutions have the authority to grant access to the data under their responsibility.

Access Management and Traceability

An access management process has been implemented to ensure traceability and control of access to the bank's data. Based on the principle of least privilege, this process is based on control through authentication and authorization, thereby increasing the level of security. The specifications for the implementation of these control measures are detailed in the latest version of the Data Security Framework in force.

Authentication establishes a list of users and restricts access by password to authorized individuals, while authorization controls access based on role and associated permissions. A register of users with the

**ACCESS LEVELS**

Access levels can be provided upon request from the principal researcher through the person responsible for the local and central coordination of the platform.

**COMMERCIALIZATION AND INTELLECTUAL PROPERTY**

**Commercialization of Research Results**

For the sake of transparency and information dissemination, it is mandatory for an institution to inform its local coordination center of any commercial valorization of a product resulting from the use of expertise and data accessible via the CODA platform. The local coordination center must also fulfill this obligation concerning participating institutions.

**Intellectual Property**

The principal researcher, their affiliated institution, and the private partner must negotiate among themselves the sharing of intellectual property rights and financial benefits.

**Dissemination of Results and Publications**

Researchers using the data must acknowledge the platform’s contribution in all types of communications and publications (written and oral) and specify the participating institutions as well as the main researchers. The acknowledgment must also be made in a multicentric context. Public disclosure on the platform’s website must be made when collaboration with the private sector occurs.

**CHANGE OF PURPOSE, SALE, MERGER, OR TRANSFER OF THE BANK**

The data may only be used for research purposes and quality improvement. Any procedure aiming to change the bank's purpose must be formally authorized by the following entities: a) the bank's steering committee; b) the affiliated institutions of the principal researchers for each subset of data accessible via the CODA platform targeted by usage outside established activities; c) the Central Coordination Center research ethics committee acting as the evaluating Research Ethics Committee.

This authorization is a requirement, and each institution has the option to withdraw its data if it does not adhere to its use for other purposes.

**COMPLIANCE**

The established structure allows restricted and limited access to projects for which ethical documentation has been granted. A dated list of projects, researchers, and users is kept for each institution by the central platform access committee.

A privacy impact assessment, largely inspired by existing models, is also carried out as needed or when a change to the management framework or policies and procedures is made by an external firm. The reference center is responsible for identifying the firm and providing feedback on the results obtained to the members of the executive committee.

Participating sites must comply with the rules of the infrastructure described in this governance framework, or risk having their access to the CODA collaborative platform disabled.

**FUNDING**

The operation of the CODA platform will involve costs related to the salary of the bank's coordination, continuous optimization of data infrastructures, data analysis, and support. For the main researchers, these costs will be charged to ongoing or future research grants. For other users, data access requests and services are subject to cost recovery from operations.
